# Letrozole ovulation regimen for frozen-thawed embryo transfer in women with polycystic ovary syndrome: study protocol for a randomized controlled trial

**DOI:** 10.1186/s13063-024-08164-z

**Published:** 2024-06-06

**Authors:** Yanqiu Xie, Min Deng, Weifen Deng, Qi Fan, Yuhua Shi

**Affiliations:** 1 Department of Reproductive Medicine, Guangdong Provincial People’s Hospital (Guangdong Academy of Medical Sciences), Southern Medical University, Guangzhou, China; 2 Reproductive Medicine Centre, Shenzhen Hengsheng Hospital, Shenzhen, China

**Keywords:** Polycystic ovary syndrome, Letrozole, Programmed regimen, Frozen-embryo transfer, Clinical pregnancy rate

## Abstract

**Background:**

Women with polycystic ovary syndrome (PCOS) are usually selected to undergo an ovulation induction regimen or a programmed regimen for endometrial preparation in the frozen-thawed embryo transfer (FET) during their IVF/ICSI treatment. The programmed regimen permits flexible scheduling of embryo transfer but requires long-term usage of exogenous estrogen and higher dosages of luteal support while the letrozole ovulation regimen needs lower dosages of luteal support only. Recently, multiple studies have shown that the letrozole ovulation regimen can improve pregnancy outcomes of FET in women with PCOS compared with the programmed regimen. However, most of these studies are retrospective, and prospective studies are urgently needed the evidence from the perspective study is insufficient.

**Methods/design:**

We are undertaking a multicentre, randomized, controlled clinical trial of an endometrial preparation regimen for FET in women with PCOS. The eligible women are randomly assigned to either the letrozole ovulation regimen or the programmed regimen for endometrial preparation. The primary outcome is the clinical pregnancy rate.

**Discussion:**

The results of this study will provide evidence for whether the letrozole ovulation regimen for endometrial preparation could improve pregnancy outcomes in PCOS women undergoing FET.

**Trial registration:**

Chinese Clinical Trial Registry ChiCTR2200062244. Registered on 31 July 2022.

**Supplementary Information:**

The online version contains supplementary material available at 10.1186/s13063-024-08164-z.

## Background

The application of frozen-thawed embryo transfer (FET) has significantly increased the live birth rate (LBR) and reduced the incidence of ovarian hyperstimulation syndrome (OHSS), especially in patients with polycystic ovary syndrome (PCOS) [1]. The ovulation induction regimen, programmed regimen, and natural regimen are the three regimens available for endometrial preparation in the frozen-thawed embryo transfer (FET). Patients with PCOS are frequently chosen to undergo an ovulation induction regimen or programmed regimen, as they typically experience irregular menstruation or ovulation disorders. The endometrium is prepared with programmed oestrogen and progesterone in the programmed regimen, allowing for flexibility in the timing of embryo transfer. However, if pregnancy is confirmed, it requires long-term use of exogenous estrogen and greater dosages of luteal support for at least 2 months. The ovulation induction regimen uses medicines such as letrozole, clomiphene, and human menopausal gonadotropin (HMG) to prepare the endometrium. The timing of embryo transfer may not be so flexible, but it only needs a lower dosage of exogenous luteal support. Even though all of these regimens are frequently used for endometrial preparation for PCOS patients, picking one over the others is still difficult.

Letrozole is a third-generation, highly specific aromatase inhibitor that decreases the concentration of estrogen in the blood by blocking the conversion of testosterone to estrogen. This alleviates the negative feedback on the pituitary and brain glands. Substantially, the pituitary gland is stimulated to secrete follicle-stimulating hormone which promotes follicle development. In recent years, letrozole has become the drug of choice for ovulation induction in FET because of its mono-ovulatory effect and its short half-life of about 48 h. Meanwhile, when letrozole alone fails to stimulate follicular growth, the timely addition of HMG can greatly reduce the incidence of cycle cancellation [2].

Several retrospective studies have found that letrozole administration in FET increased clinical pregnancy rate (CPR) and LBR [3–6] and decreased the incidence of hypertensive disorders of pregnancy in women with PCOS compared to the programmed regimen [7]. However, a single-centre, randomized, controlled clinical trial found no significant difference between the two regimens in terms of CPR and embryo implantation rate [8]. According to a recent meta-analysis, the ovulation induction regimen increased the LBR and reduced the risk of miscarriage, preeclampsia, and preterm labour in patients with PCOS when compared to the programmed regimen. In the subgroup analysis, on the other hand, they evaluated different ovulation regimens and found that the letrozole ovulation regimen achieved a higher CPR [9]. Therefore, further prospective research is required to confirm whether the letrozole ovulation regimen can achieve better pregnancy outcomes than the programmed regimen.

This study is a multi-centre randomized controlled clinical trial. It compares the efficacy and safety of the letrozole ovulation regimen with the programmed regimen in women with PCOS undergoing FET.

## Methods/design

### Design and setting

This study is a multicentre, randomized controlled, open-label clinical trial to evaluate whether the letrozole ovulation regimen for endometrial preparation can improve the clinical pregnancy rate in PCOS patients as compared to the programmed regimen. Patients are recruited from six hospitals, including Guangdong Provincial People’s Hospital, General Hospital of Ningxia Medical University, the Second Hospital of Hebei Medical University, Xiamen Maternal and Child Health Care Hospital, Hospital for Reproductive Medicine Affiliated to Shandong University, and Xiangya Hospital, Central South University, spreading across different Chinese provinces—Guangdong, Shandong, Fujian, Hebei, Hunan, and Ningxia Autonomous Region.

### Inclusion criteria

The inclusion criteria are as follows:

1. Women diagnosed with PCOS according to the Rotterdam criteria [10]
2. Women aged under 38 years
3. Frozen embryos from the first and/or second oocyte retrieval cycles

### Exclusion criteria

The exclusion criteria are as follows:

1. Women with a history of unilateral or bilateral ovarian surgery.
2. Women with a history of recurrent spontaneous abortion.
3. Women with a uterine cavity abnormality, such as double uterus, uterine septum, and moderate and severe intrauterine adhesion.
4. Chromosome abnormality (except chromosomal polymorphism) of either partner.
5. A preimplantation genetic test is required.

**Fig. 1:**
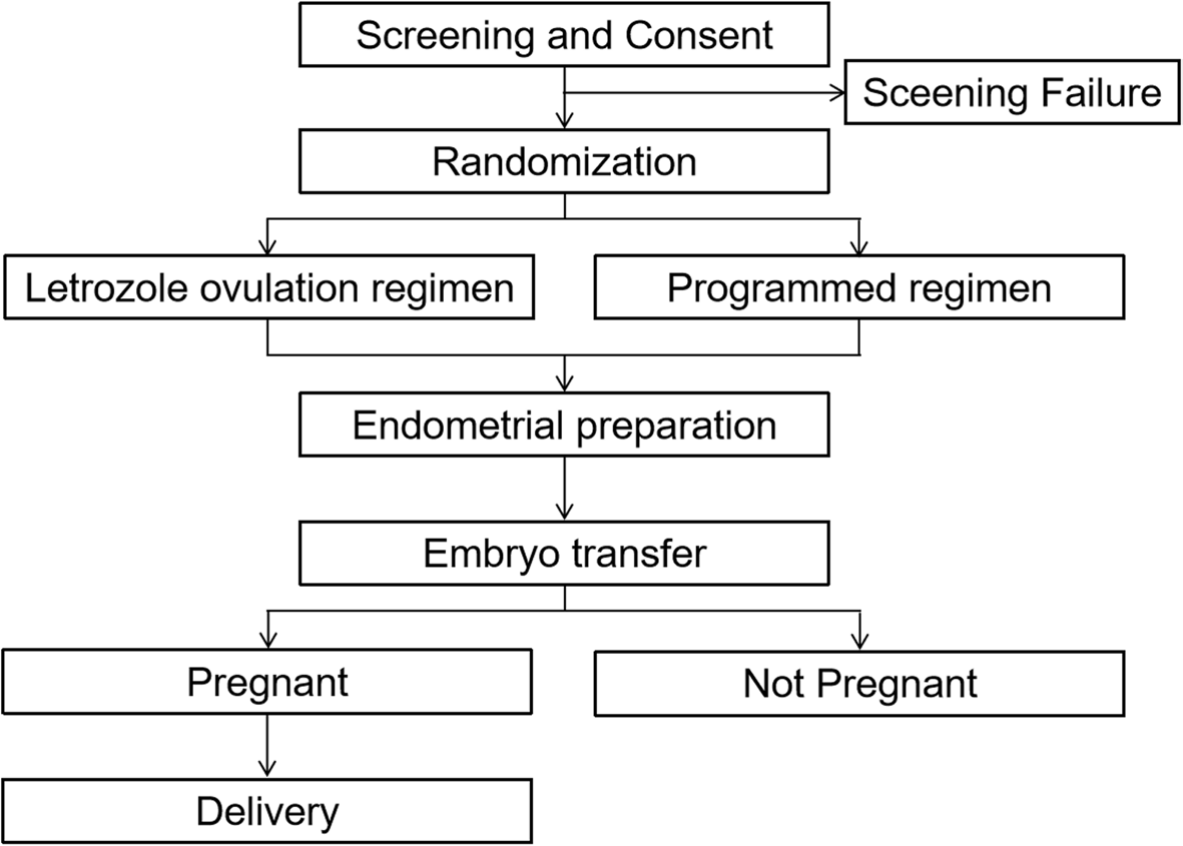
Study flowchart

The patients will be randomized (1:1) to the letrozole ovulation regimen group or the programmed regimen group in the FET cycle, and their pregnancy and perinatal outcomes during this cycle will be followed up and analysed. The flowchart will follow the study protocol as shown in Fig. 1. The items of this trial can be found in the WHO Trial Registration Data Set as shown in Additional file 1.

### Sample size

The PASS software version 15.0.5 (NCSS, LLC, Kaysville, UT, USA) was used to estimate the sample size. We assumed that the clinical pregnancy rate is 65% in the letrozole ovulation regimen group and 40.8% in the programmed regimen group based on past retrospective investigations [6]. To detect an absolute difference in CPRs, the study will involve 66 women in each group with a power of 80% and an alpha error of 0.05. Considering a 10% follow-up drop-out rate, our target sample size will be 148 women.

To minimize the loss of follow-up, we optimize our strategies of follow-up, including text messaging reminders to patients’ mobile phones the day before scheduled appointments, reducing the number of clinic visits if possible, and regular review of patients with missed appointments to allow early contact and intervention.

### Screening

**Fig. 2:**
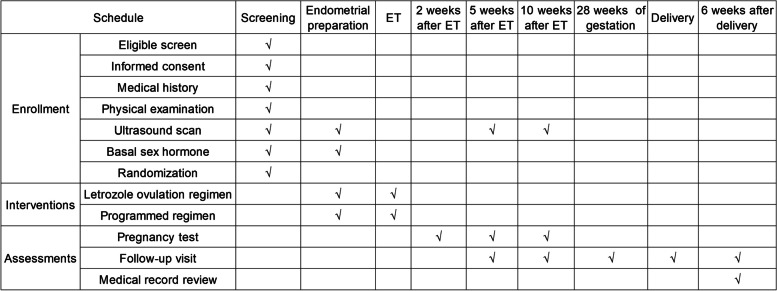
SPIRIT diagram for the schedule of enrollment, interventions, and assessments. ET, embryo transfer

We advertise for clinical study patient recruitment. PCOS patients who intend to undergo frozen embryo transfer will be screened according to inclusion and exclusion criteria. The eligible patients will sign informed consent after the assessment. The standardized case report forms are used to capture current medication status and previous medical history. Patients’ names are replaced with their phonetic initials to protect their privacy. A physical examination (height, body weight, waistline, hipline, blood pressure) and transvaginal ultrasound scan are performed. A diagnostic hysteroscopy is conducted on patients who are suspected of having an abnormal uterine cavity after an ultrasound scan. The serum basal sex hormones, anti-Müllerian hormone, glucose and lipid levels, and renal and hepatic function tested in the recent half year will be recorded. The research protocol, version 1.1, was finalized on May 30, 2022. The Standard Protocol Items: Recommendations for Interventional Trials (SPIRIT) figure (Fig. 2) shows the schedule for enrollment, interventions, and evaluation. The SPIRIT checklist is presented in Additional file 2.

### Randomization and blinding

A computer-generated random list is generated by SPSS 16.0 (SPSS Inc., Chicago, IL, USA). The statisticians will upload the random list into the online database’s central grouping system and maintain the random list absolutely confidential. The list is not accessible to the researchers. They are only given the group number when a patient is enrolled. Letrozole tablets or estradiol valerate tablets will be given to participants. Since the trial is open-label, neither the participants nor the physicians are blind to the group information, while all the generated data in the trial will be analysed by blinded independent biostatisticians.

### Interventions

#### Endometrial preparation

During menstruation, the endometrial preparation can begin if the follicles are less than 10 mm, and serum estradiol and progesterone are at the basic levels. Eligible patients are assigned at random to either the letrozole ovulation regimen or the programmed regimen.

Within 5 days of the menstrual cycle, women in the letrozole ovulation group will receive 2.5 or 5.0 mg of letrozole each day for 5 days. Around the 12th day of menstruation, serum hormone levels and ultrasound monitoring will be carried out. If there is no dominant follicle, low-dose HMG injections will be used to promote follicle growth. Human chorionic gonadotropin (HCG) ampules will be injected intramuscularly for the final oocyte triggering when the diameter of the dominant follicle measures at least 17 mm and the thickness of the endometrium measures at least 7 mm.

Within 3 days of menstruation, women in the programmed regimen group will take tablets containing 4–8 mg of estradiol valerate daily. After 10 days, an ultrasound will be used to determine the thickness of the endometrium and serum sex hormones will be measured. It is appropriate for shifting the endometrium when its thickness reaches 7 mm by administering dydrogesterone, Utrogestan, or Crinone either alone or in combination.

The FET cycle will be cancelled if the thickness of the endometrium in either group is less than 7 mm or if there is no follicle growth in the letrozole ovulation group even after the addition of HMG.

#### Embryo transfer and luteal support

A maximum of two blastocysts or embryos in the cleavage stage could be transferred. An experienced physician will carry out the embryo transfer, with trans-abdominal ultrasonography serving as guidance.

For luteal support, dydrogesterone 20 mg PO bid, Utrogestan 200 mg pv bid, and Crinone 90 mg pv qd are the most commonly prescribed medications. For patients in the letrozole ovulation group, one of them will be used starting on the day of ovulation. The transfer timing of frozen embryos is determined based on the time of ovulation. On the third day after ovulation, the D3 embryo will be transferred, and on the fifth day, the D5 embryo.

One or two drugs for luteal support will be administered to the patients in the programmed regimen group. If the pregnancy is confirmed, the use of luteal support and estradiol valerate tablets should be continued until 7–8 weeks of gestation, after which their dosages should be gradually decreased.

### Outcome and outcome assessments

The primary outcome is the clinical pregnancy rate, defined as the ratio of the clinical pregnancy (intrauterine gestation sac) number to the number of patients who received embryo transfer. The secondary outcomes include abortion rate, live birth rate, birth weight, pregnancy and perinatal complication, and neonatal complication.

Two weeks following embryo transfer, serum β-HCG is tested; a result of ≥ 10 IU/L indicates biochemical pregnancy. The patients who are biochemically pregnant will keep taking medications for luteal support. The existence of a gestational sac within the uterine cavity is referred to as clinical pregnancy. When the embryo implants anywhere other than the uterine cavity, it is referred to as an ectopic pregnancy. Around 5 weeks following embryo transfer, a transvaginal ultrasound is carried out to confirm clinical pregnancy. When a viable fetus with a fetal heartbeat is detected by ultrasound around 10 weeks following embryo transfer, it is considered to be an ongoing pregnancy.

Abortion rate is defined as the ratio of the number of nonviable pregnancies before 28 weeks of gestation to the number of clinical pregnancies. Abortion before 12 weeks of pregnancy is called early abortion while after 12 weeks of pregnancy is called late abortion. Live birth rate refers to the ratio of the number of normal fetuses delivered at 28 weeks or later of gestation to the number of patients who received embryo transfer. Preterm birth occurs between 28 and 37 weeks of gestation. A birth at 37 weeks or later is called a full-term birth. The congenital malformations are diagnosed after birth.

### Follow-up plan

There are four follow-up visits via medical records or telephone calls if women achieve clinical pregnancy.

Around 13 weeks of gestation, information on the ongoing pregnancy and any first-trimester pregnancy complications, such as early abortion and ectopic pregnancy, will be collected.

Around 29 weeks gestation, the second-trimester pregnancy complications—such as hypertensive disorders of pregnancy, gestational diabetes mellitus, and late abortion—will be recorded.

At the time of delivery, information on obstetrical and perinatal complications, as well as neonatal medical records, is acquired.

The final visit follows the postpartum complications and neonatal complications 6 weeks after delivery.

Timely follow-up visits should be conducted to reduce the number of participants who discontinue or deviate from intervention protocols.

Women will be asked about adverse events at each visit. Adverse events (AEs) are defined as any adverse medical problems that arise during the research period, without regard to their connection to the intervention. AEs in this study will be collected systemically and divided into study-related AEs and non-study-related AEs, which will be judged by clinicians at the time of recording every clinic visit.

All AEs will be recorded in detail and reported. Once any adverse reaction occurs during medication use, the drug will be stopped immediately. Serious AEs will be reported to the principal investigator immediately, and appropriate measures will be initiated instantly. The ethics committee will determine whether the AE is likely to have been associated with the experimental drug. The following conditions are considered serious AEs: death, life-threatening, severe or permanent disability, congenital malformation or birth defect, neonatal death within 6 weeks of delivery, or any other event that the local principal investigators deem serious. Adverse events must be timely noted and reported.

### Data management

The data originates mainly from medical records and follow-up records. We use an electronic data capture (EDC) system to record and deposit the study data. Researchers will verify the original data and then data entry staff will enter it into the EDC after receiving professional training, which significantly ensures the accuracy of the input data. Consent forms, screening and identification records, and other participant-identifiable data will all be stored in site files and only researchers with permission can be able to access them. Paper files will be kept in a locked filing cabinet in the treating hospital. Electronic documents will be stored in a password-protected computer, with access restricted to the principal investigator. All research documents will be preserved for at least 5 years after publication.

### Independent Data Monitoring Committee (DMC)

An independent DMC has been appointed for this trial to oversee the safety monitoring. The DMC will review on a regular basis accumulating data from the ongoing trial and monitor the continuing safety of current participants and those yet to be recruited, as well as reviewing the validity and scientific merit of the trial.

The DMC composition, name, title, and address of the chairman and of each member are given in the DMC Charter which is in line with that proposed by the DAMOCLES Study Group [11]. Membership includes expertise in the relevant field of study, statistics, and research study design.

The DMC Charter includes, but is not limited to, defining the following: (1) the schedule and format of the DMC meetings, (2) the format for the presentation of data, (3) the method and timing of providing interim reports, (4) stopping rules.

The DMC is independent of the sponsor, Ethics Committees, regulatory agencies, investigators, clinical care of the trial participants, and any other capacity related to trial operations. The DMC has the responsibility for deciding whether, whilst randomization is in progress, the unblinded results (or the unblinded results for a particular subgroup) should be revealed to the researchers. The DMC Charter states that they will do this if, and only if, two conditions are satisfied: (1) the results provide proof beyond reasonable doubt that treatment is on balance either definitely harmful or definitely favourable for all, or for a particular category of, participants in terms of the major outcome and (2) the results, if revealed, would be expected to substantially change the prescribing patterns of clinicians who are already familiar with any other trial results that exist. Exact criteria for ‘proof beyond reasonable doubt’ are not, and cannot be, specified by a purely mathematical stopping rule, but they are strongly influenced by such rules. The DMC Charter is in agreement with the Peto-Haybittle [11, 12] stopping rule whereby an interim analysis of a major endpoint would generally need to involve a difference between treatment and control of at least three standard errors to justify premature disclosure. An interim subgroup analysis would, of course, have to be even more extreme to justify disclosure. This rule has the advantage that the exact number and timing of interim analyses need not be pre-specified. In summary, the stopping rules require extreme differences to justify premature disclosure and involve an appropriate combination of mathematical stopping rules and scientific judgement.

### Data analysis

All the generated data in the trial will be analysed by blinded independent biostatisticians. The primary analysis will be performed according to the intention-to-treat (ITT) principle. The Statistical Package for the Social Sciences (version 24.0; SPSS Inc., USA) will be used for all analyses. All tests will be two-tailed, and a *P* value < 0.05 is considered to be statistically significant. The primary outcome, clinical pregnancy rate, will be analysed using the chi-square test. The secondary outcomes such as abortion rate, live birth rate, and some complications will be analysed using the chi-square test or Fisher’s exact test for expected frequencies less than 5. The other parameters will be analysed and compared according to the following methods. The Shapiro-Wilk test will be used to test the normal distribution of continuous variables. For normally distributed data, we will use Student’s *t*-test to examine the differences between groups; for non-normally distributed data, we will use the Wilcoxon rank-sum test. Continuous variables will be represented as means ± standard deviations. Categorical variables will be described as frequencies or percentages, and the chi-square test or Fisher’s exact test is used to examine the differences between groups.

We will perform a secondary per-protocol analysis according to the actual treatment that subjects received who comply with the protocol. The data analysis methods are as above.

Missing data will be treated as missing at random and will be imputed using the last observation carried forward method. For the missing values, a sensitivity analysis will be done under the hypothesis of the worst and the best outcomes for each missing individual. Therefore, all secondary per-protocol analysis outcomes will be considered exploratory. All statistical analyses will be done using the statistical package SPSS (version 24.0; SPSS Inc., USA).

### Plans for communicating important protocol amendments to relevant parties, including trial participants, ethical committees, and so on

According to the Medicines for Human Use (Clinical Trials) Regulations 2004, the Directive 2001/20/EC, and Clinical Trial Regulation EU No. 536/2014, the Investigator may make a substantial amendment at any time during a clinical trial. Substantial amendments will not be implemented at the site until the concerned Ethics Committee has approved, except where necessary to eliminate an immediate hazard to a trial patient or when the change(s) involves only logistical or administrative aspects of the trial. Any other deviation from the protocol that has not been approved by the Investigator and the Ethics Committee could result in discontinuation from the study of the centre involved.

A substantial amendment is defined as an amendment to the terms of the application, or to the protocol or any other supporting documentation, that is likely to affect to a significant degree:

- The safety or physical or mental integrity of the infants of the study
- The scientific value of the study
- The quality and safety of study treatment
- The conductor management of the study

In these cases, a written amendment will follow the regulations in place. All substantial amendments will be notified to the Ethics Committee and the Competent Authority. Non-substantial amendments will not be notified to the Ethics Committee and the Competent Authority but will be recorded and filed by the Investigator.

All investigators participating in the study must be aware of any protocol amendments and must respect their content.

## Discussion

This study will compare the efficacy and safety of the letrozole ovulation regimen with the programmed regimen in women with PCOS undergoing FET. We intend to enrol at least 148 subjects from 6 academic IVF centres in China. Approximately half of the subjects have been enrolled by the time of manuscript preparation since enrollment started in September 2022. The result of this multi-centre randomized trial will provide strong evidence for the strategy of FET in patients with PCOS.

Recently, a freeze-all and delayed FET policy has gained widespread support [13–15]. Our previous study demonstrated that FET contributed to a higher rate of live birth, a lower incidence of OHSS, and a higher birth weight of the singleton in patients with PCOS when compared to fresh embryo transfer [1]. The reason for a higher success rate of FET may be that the endometrium is typically spared from the adverse effects of supraphysiological concentration of estrogen.

The first choice of endometrial preparation in FET for PCOS patients is still controversial. Women who use the programmed regimen have a lower embryo implantation rate and higher incidence of caesarean delivery, preeclampsia, and postpartum haemorrhage in contrast to the natural regimen [16, 17]. These outcomes could be attributed to the application of high-dose exogenous estrogen and a lack of endogenous luteal support. According to a retrospective study conducted in America, LBR was comparable between the letrozole FET group and the programmed FET group among anovulatory women [18]. However, a Chinese retrospective study indicated that the ovulation induction regimen would be the better choice [19]. Furthermore, the letrozole ovulation regimen could reduce the incidence of hypertensive disorders of pregnancy, gestational diabetes mellitus, and large for gestational age infants in patients with PCOS [20–22]. These benefits may be related to promoting the expression of integrins, leukaemia inhibitory factor, L-selectin, and the formation of cytosolic convexity induced by letrozole, which improves endometrial tolerance and helps patients achieve better pregnancy outcomes [23, 24].

Whether the letrozole ovulation regimen could achieve better pregnancy outcomes in PCOS patients is of great clinical significance. This study is expected to provide a reliable answer.

## Trial status

The enrollment is ongoing at the time of manuscript submission. Recruitment for the first patient was started in September 2022. The estimated end date of the last recruitment in this study is December 2024. The research protocol, version 1.1, was completed on May 30, 2022.

### Supplementary Information

Additional file 1: WHO Trial Registration Data Set.

Additional file 2: SPIRIT checklist.

Additional file 3: Composition of the independent Data Monitoring Committee (DMC).

Additional file 4: Inform and consent.

## Data Availability

The datasets generated during the current study are available from the corresponding author upon reasonable request. The trial results will be publicated after finishing all visits.

## Abbreviations

AEs: Adverse events
CPR: Clinical pregnancy rate
DMC: Data Monitoring Committee
EDC: Electronic data capture
ET: Embryo transfer
FET: Frozen-thawed embryo transfer
HCG: Human chorionic gonadotropin
HMG: Human menopausal gonadotropin
LBR: Live birth rate
OHSS: Ovarian hyperstimulation syndrome
PCOS: Polycystic ovary syndrome
