# Correction: Letrozole ovulation regimen for frozen-thawed embryo transfer in women with polycystic ovary syndrome: study protocol for a randomized controlled trial

**DOI:** 10.1186/s13063-024-08319-y

**Published:** 2024-07-09

**Authors:** Yanqiu Xie, Min Deng, Weifen Deng, Qi Fan, Yuhua Shi

**Affiliations:** 1 Department of Reproductive Medicine, Guangdong Provincial People’s Hospital (Guangdong Academy of Medical Sciences), Southern Medical University, Guangzhou, China; 2 Reproductive Medicine Centre, Shenzhen Hengsheng Hospital, Shenzhen, China

**Correction: Trials 25, 401 (2023)**

**https://doi.org/10.1186/s13063-024-08164-z**

Following the publication of the original article [1], we were notified that the first letter of every keyword was accidentally removed during article processing.

Originally published keywords were: Olycystic ovary syndrome, Etrozole, Rogrammed regimen, Rozen-embryo transfer, Linical pregnancy rate.

Correct keywords: Polycystic ovary syndrome, Letrozole, Programmed regimen, Frozen-embryo transfer, Clinical pregnancy rate.

The original article was corrected.
