# Supplementary material for: Letrozole ovulation regimen for frozen-thawed embryo transfer in women with polycystic ovary syndrome: study protocol for a randomized controlled trial
Source: Trials. 2024 Jun 6;25:364. doi: 10.1186/s13063-024-08164-z (PMC11155015; doi:10.1186/s13063-024-08164-z)
Supplement: Supplementary file 1 — Additional file 1: WHO Trial Registration Data Set. [file 13063_2024_8164_MOESM1_ESM.pdf]

Table.1 WHO Trial Registration Data Set

Main Information

Register:	ChiCTR
Last refreshed on:	3-Apr-23
Main ID:	ChiCTR2200062244
Date of registration:	2022/7/31
Prospective Registration:	Yes
Primary sponsor:	Guangdong Provincial People's Hospital
Public title:	Randomized controlled trial of letrozole use during frozen embryo transfer cycles in women with polycystic ovary syndrome
Scientific title:	Randomized controlled trial of letrozole use during frozen embryo transfer cycles in women with polycystic ovary syndrome
Date of first enrolment:	2022/8/1
Target sample size:	Experimental group:100;Control group:100;
Recruitment status:	Recruiting
URL:	http://www.chictr.org.cn/showproj.aspx?proj=173844
Study type:	Interventional study
Study design:	Parallel
Phase:	Parallel
Countries of recruitment	China

Contacts

Name:	Xie Yanqiu
Address:	106 Second Zhongshan Road, Yuexiu District, Guangzhou, Guangdong [illegible]
Telephone:	+86 15902037908
Email:	xxyyqq7890@163.com
Affiliation:	Guangdong Provincial People's Hospital
Name:	Shi Yuhua
Address:	106 Second Zhongshan Road, Yuexiu District, Guangzhou, Guangdong 510000
Telephone:	+86 18615177822
Email:	shiyuhua2003@126.com
Affiliation:	Guangdong Provincial People's Hospital

Key inclusion & exclusion criteria

Inclusion criteria: 1. According to the Rotterdam diagnostic criteria for PCOS, 2 of the
(1) Sparse or anovulatory ovulation;
(2) Clinical manifestations of hyperandrogen and/or hyperandrogenemia;
(3) Polycystic ovaries: Ultrasound indicated >=12 follicles in one or both ovaries with a diameter of 2-9mm and/or >=10ml ovarian volume.

2. The woman aged <= 38 years.
3. IVF/ICSI frozen embryo transplantation <=2 ovulation cycles.
Exclusion criteria: 1. History of surgery on one or both ovaries;
2. History of recurrent abortion;
3. Untreated uterine malformations or abnormalities: double uterus, mediastinal uterus
4. Chromosome abnormalities in one or both parties (except chromosome polymorphism);
5. Preimplantation genetic screening is required.

Age minimum: 20
Age maximum: 38
Gender: Female

Health Condition(s) or Problem(s) studied

polycystic ovary syndrome (PCOS)

Intervention(s)

Experimental group:Letrozole;Control group:Estradiol;

Primary Outcome(s)

clinical pregnancy rate

Secondary Outcome(s)

abortion rate, live birth rate, birth weight, pregnancy and perinatal complication, and neonatal

Source(s) of Monetary Support

National Key R&D Program of China (2021YFC2700404)
