# Supplementary material for: Letrozole ovulation regimen for frozen-thawed embryo transfer in women with polycystic ovary syndrome: study protocol for a randomized controlled trial
Source: Trials. 2024 Jun 6;25:364. doi: 10.1186/s13063-024-08164-z (PMC11155015; doi:10.1186/s13063-024-08164-z)
Supplement: Supplementary file 3 — Additional file 3: Composition of the independent Data Monitoring Committee (DMC). [file 13063_2024_8164_MOESM3_ESM.pdf]

Table.2 Composition of the independent Data Monitoring Committee (DMC)

Name	Affiliation	Expertise
Zhang,Jun	Xinhua Hospital, Shanghai Jiaotong University School of Medicine	Statistician（Chairman）
Gao,Qin	Center for Reproductive Medicine, Shandong University	Trials expert
Wang,Ze	Center for Reproductive Medicine, Shandong University	Statistician
Han,Ting	Center for Reproductive Medicine, Shandong University	Clinical expert
Lu,Qun	Beijing Chaoyang Hospital Affiliated to Capital Medical University	Clinical expert
