# Supplementary material for: Letrozole ovulation regimen for frozen-thawed embryo transfer in women with polycystic ovary syndrome: study protocol for a randomized controlled trial
Source: Trials. 2024 Jun 6;25:364. doi: 10.1186/s13063-024-08164-z (PMC11155015; doi:10.1186/s13063-024-08164-z)
Supplement: Supplementary file 4 — Additional file 4: Inform and consent. [file 13063_2024_8164_MOESM4_ESM.pdf]

知情同意书

项目名称：来曲唑方案在多囊卵巢综合征患者冻胚移植中的临床研究（LE-PCOS-FET）

我们诚邀你参加这项临床研究，研究题目如上，你需要决定是否愿意参加，做出决定前请慢慢考虑，请仔细阅读下文内容，你有任何问题都可以询问医生。

为何要进行该项研究?

随着玻璃化胚胎冷冻技术的推广，选择性单胚胎移植及全胚冷冻理念的提出，越来越多的胚胎选择冷冻保存。针对多囊卵巢综合征（polycystic ovary syndrome, PCOS）患者，冻融胚胎移植（frozen thawed embryo transfer, FET）可以提高活产率、降低流产率和卵巢过度刺激综合征的发生。关于PCOS最合适的内膜准备方案，目前尚无定论。多篇回顾性研究发现在PCOS患者中，来曲唑（letrozole，LE）促排卵方案相较于人工周期有更好的助孕结局，可以增加胚胎种植率、临床妊娠率及继续妊娠率，提高患者的活产率，降低流产率。也有单中心小样本的随机对照临床试验研究发现LE促排卵方案与人工周期两组间的胚胎种植率、生化妊娠率、临床妊娠率无明显统计学差异。因此，PCOS患者FET使用LE促排卵进行内膜准备是否能获得更好的助孕结局仍存争议，尚缺乏大样本多中心的前瞻性临床研究。

本项研究的目的是评估在PCOS患者FET中使用LE促排卵方案进行内膜准备是否可以改善患者的临床妊娠率，降低流产率。

全国将有6家医院的生殖中心参加本研究，约200例PCOS进行FET被纳入。

哪些患者将会被参加该项研究？

你被邀请参加本研究，是因为你有下述情况：

根据鹿特丹标准诊断PCOS：

1.以下3项中符合2项并排除其它高雄激素的病因。

（1）稀发排卵或无排卵；

（2）高雄激素的临床表现和/或高雄激素血症；

（3）多囊卵巢：超声提示一侧或双侧卵巢直径 2-9mm 的卵泡≥12 个和/或卵巢体积≥10ml。

2. 女方年龄≤38 岁；

3. ≤2 个取卵周期的 IVF/ICSI 进行的冻胚移植。

如果你有下述情况，你将不能参加该项研究：

1. 单侧或双侧卵巢手术史；
2. 复发性流产病史；
3. 未经治疗的子宫畸形或异常：双子宫、纵隔子宫（完全或不完全）、中重度宫腔粘连等；
4. 一方或双方染色体异常（除外染色体多态性）；
5. 需进行胚胎植入前遗传学筛查。

参加该项研究，你需要干什么？

如果你被纳入本研究，你所接受的所有治疗都是临床上常规进行的治疗措施，与通常冻融胚胎移植唯一不同的是在该项研究中将有 2 组患者，在进行 FET 内膜准备前你将会被随机分配到两组中的一组，一组用来曲唑准备内膜，另外一组用人工周期准备内膜；如果你实现了临床妊娠，妊娠期间我们会关注孕期并发症的随访，分娩后会询问你分娩的相关信息。

参加该项研究需要多久？

治疗过程从 FET 内膜准备开始，内膜达标后行冻胚移植，如果你实现了临床妊娠，对妊娠期以及产后的随访约 1 年。因此从你被纳入该研究至研究结束，大约需要 12 个月时间。

参加该项研究会有什么风险和副反应？

与通常的接受冻融胚胎移植的患者比较，参加该项研究不会增加额外风险。通常的冻融胚胎移植技术中可能的风险和不良反应都在体外受精胚胎移植、胚胎冷冻等的知情同意书中有详细的说明。内膜准备中可能使用来曲唑诱发排卵，来曲唑常见的副作用为潮热、关节痛、恶心和疲劳，但由于使用时间短、剂量小，发生风险较低，目前研究认为使用来曲唑进行促排卵并不会增加子代先天畸形的

风险。

本研究将排除存在“试管婴儿”或者妊娠禁忌症的患者。胚胎移植过程将由有资质而且有经验的医生完成。每个分中心都将告知患者研究者电话或者值 24 小时班的住院医师的电话，一旦患者发生不良事件可以联系到我们。本研究将记录所有患者发生的不良事件，包括严重不良事件。数据与安全监察委员会将每 6 个月对一般不良反应记录进行评估 1 次，而对于严重的不良反应则会及时进行评估。

本研究将尽最大努力来减少对参与者造成的损伤，但是不能排除参加本研究会发生并发症或者损伤的可能。

参加本研究会有什么好处？

本研究所采用的治疗方案均为临床常用治疗方案，药物、检查费用与常规诊疗一样，但参加本研究会得到优先待遇，尽可能减少就诊等待时间等。你将被随机分配到任一治疗组，最后的研究结果可能表明你接受的治疗比另外一组及其他治疗方法更有效或者不良反应的发生率更低。本研究的结果对将来与你有同样情况的患者的治疗具有指导价值，并将有助于促进医学界对 PCOS 患者 FET 内膜准备方案选择的认识。

你可以拒绝参加该项研究吗？

参加该项研究是自愿的，你可以拒绝参加。如果你决定参与该项研究，你需要签署书面的知情同意书。在研究期间你可以要求退出本研究，你的退出将不会受到任何影响。如果你想退出研究，请告知你的医生，在你退出之前收集的数据仍将用于结果分析。

如果你在参加研究的过程中发生了不良反应而继续参加本研究可能会影响你的健康，或者你没有按照本研究的要求去做，研究者有可能在未获得你同意的情况下要求你退出。如果你在研究结束之前退出研究，你需要在你退出的时候告知研究者。

在你参加该研究期间，我们会告知你与你所患疾病相关的医学研究进展，了解这些进展后，你可以决定是否继续参加本研究。

隐私与保密

本研究将严格执行严格的隐私保护政策，所有与本单位的通信联系都将被妥善保管，任何能识别你的个人信息都不会因为任何理由而泄露给本单位以外的任何人。即使本研究的结果被发表，你的个人信息也不会被公开。

你有权利了解你的个人信息和最终的研究结果。你的个人信息在数据收集、保存及应用（包括分析和比较）的过程中都将受到隐私保护。

该研究的联系方式

你有权询问关于本研究的任何问题。如果你有疑问、顾虑或者你认为自己发生了不良反应，工作时间请打电话__________，非工作时间请打这个电话__________联系我们。

同意声明

我已经阅读了（或者研究者已经向我介绍了）参加本研究的相关信息，我有充足的时间考虑是否参加该研究。研究者对我的疑问给予了满意的回答。我同意参加该研究，同意该项研究的研究人员使用我的病历记录中的数据。

我知道:

1．这是一项临床研究，目的是评估在PCOS患者FET中使用LE促排卵方案进行内膜准备是否可以改善患者的临床妊娠率，降低流产率。

2. 我的身份及可以识别我的信息都将被保密。

3. 我参加这项研究是自愿的，我可以退出研究，而我的治疗将不受到任何影响。

4. 我可以向医生或者研究者询问任何关于该研究的信息。

在下面签名表示你已经理解了上述信息，已经对疑问之处做了询问并得到了回答。

患者签字______________ 日期_________________ （签字要清晰）

研究人员：在下面签名表明你已经向该患者介绍了该项研究，并回答了患者的对于该研究的疑问(仅本研究批准的研究人员可以向患者介绍本研究并获得知情同意)。

研究人员签字______________ 日期 _________________（签字要清晰）
